# Editorial: Infectious Agent-Induced Chronic Immune Activation: Causes, Phenotypes, and Consequences

**DOI:** 10.3389/fimmu.2021.740556

**Published:** 2021-12-10

**Authors:** Caroline Petitdemange, Nicholas Funderburg, John Zaunders, Pierre Corbeau

**Affiliations:** ^1^ Institut Pasteur, HIV Inflammation and Persistence Unit, Paris, France; ^2^ School of Health and Rehabilitation Sciences, Ohio State University, Columbus, OH, United States; ^3^ St Vincent’s Centre for Applied Medical Research, St Vincent’s Hospital, Sydney, NSW, Australia; ^4^ CHU de Nîmes, Institut de Génétique Humaine CNRS-Université de Montpellier, UMR9002, Montpellier, France

**Keywords:** inflammation, comorbidity, pathogen, immune cell, immune system

## Summary

Persistent immune activation and dysfunction induced by infectious agents may contribute to long term comorbid conditions in individuals exposed to these pathogens. Even during successful antimicrobial treatment, increased levels of inflammation and immune cell activation and inappropriate immune cell migration and retention in tissue sites may contribute to tissue damage and end organ disease (e.g., atherosclerosis or liver or kidney damage). Understanding why some populations infected with a pathogen are able to regulate their immune responses and limit their inflammatory consequences, while other individuals may have exacerbated and persistent immune responses even during suppressive therapy, may provide insights for development of complementary immune-modulatory therapies that may reduce inflammation and morbidity and mortality in these groups. The Research Topic highlights some of these issues.

Any infection induces the activation of the human immune system *via* various mechanisms. The problem is that although some forms of this immune activation are beneficial for the eradication of the microbial agent, others may be toxic, not only in the short term but also in the long term. It is therefore important to understand these pathogenic pathways, inasmuch as they may persist even under efficient antimicrobial therapy.

## The Ways an Infectious Agent May Trigger the Immune System

**Figure 1:**
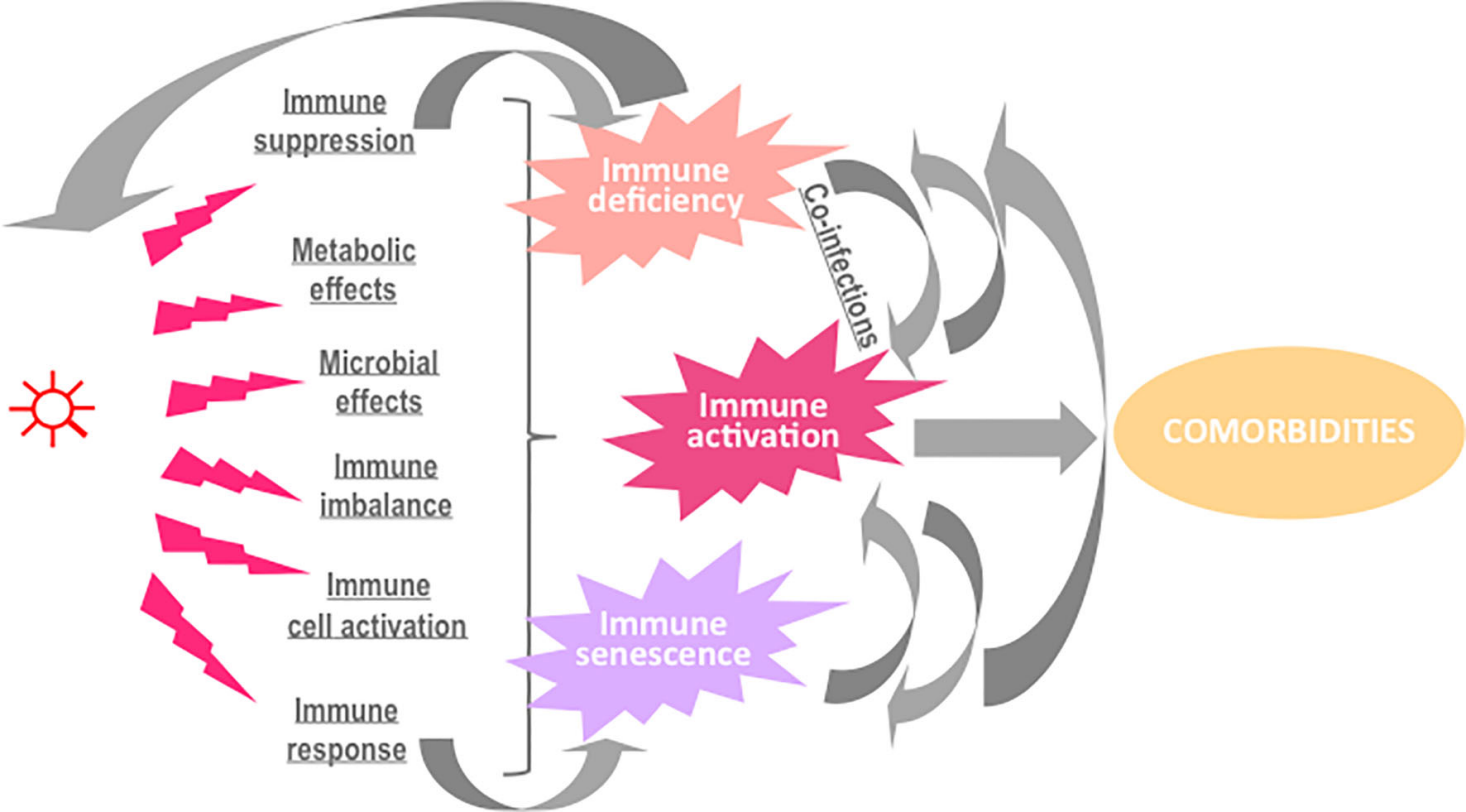
Potential causes and consequences of immune activation in the course of an infection.

Some microbial components may activate the complement and/or the coagulation system and thereby start an inflammatory reaction. Phagocytic cells will be recruited into infected tissues, and activated to engulf the infectious agent in order to neutralize it. In the meantime, immune and non-immune cells detect the presence of pathogen-associated molecular patterns, triggering a danger signal that also fuels inflammation and promoting the initiation of the adaptive immune response, ideally, resulting in a cellular and humoral immune response targeting specific microbial antigens. Natural killer cells and cytotoxic T cells will identify infected cells and destroy them. All of these types of immune activation may contribute to the elimination of the invader. Yet, besides these desirable effects, the microbial agent may also cause side effects with potential pathogenic consequences (**Figure 1**).

First, many infectious agents have developed the means to evade defenses and subsequently promote immune dysfunction. A master of this gameplay is the human cytomegalovirus (HCMV). HCMV downregulates the density of HLA classes I and II at the surface of the cells it infects (1) and interferes with their ability to present antigens (2), reducing thereby CD4+ T cell and CD8+ T cell specific responses. To prevent the attack by natural killer cells as a consequence of HLA I downregulation, the virus encodes a nonfunctional class I MHC homolog as a decoy (3). Other homologs encoded by HCMV are chemokine receptor and Fc receptor homologs acting as sinks for local chemokines and antibodies, respectively (4, 5). HCMV also inhibits macrophage motility by downregulating chemokine receptors, inducing the production of macrophage migration inhibitory factor, and acting directly on macrophage cytoskeleton functionality (6). In addition, this herpesvirus reduces complement activation by inducing natural complement inhibitors (7), and inflammation by coding for a viral form of the anti-inflammatory cytokine IL-10 (8). Finally, HCMV reduces the ability of T cells to proliferate and to secrete IL-2 and IFN-γ (9). The infectious agent itself will benefit from the immune deficiency it has induced, but other infections may also be thereby boosted, promoting a cycle of more infections and more immune activation and dysfunction.

Second, infections may indirectly activate the immune system *via* metabolic disorders. Thus, Human Immunodeficiency Virus (HIV) infection increases the level of oxidized LDL able to activate monocytes *via* Lectin-like oxidized LDL receptor-1 (10). Proinflammatory lipid classes and species (including ceramides and saturated fatty acids), the levels of which are altered by HIV-1 and antiretroviral therapy (ART), may also promote inflammation and cardiometabolic complications (11, 12). Infections may also indirectly activate the immune system through microbial disorders. For instance, influenza virus has been shown to favor bacterial species in the gut lumen (13). In addition to modifying the microbiota, HIV facilitates the translocation of microbial components from the gut lumen into the circulation by depleting Th17 cells in the gut-associated lymphoid tissue and by causing a local inflammation (14). The same is true for HCMV, known to infect gut epithelial cells and to provoke microbial translocation (15).

Third, some infectious agents may perpetuate immune activation by altering immune suppressor cell function. As an example, HIV has been reported to create a quantitative and probably qualitative deficiency in regulatory T cells (16).

Fourth, microbial components often directly activate immune cells. HIV is again a good example of this; HIV RNA stimulates plasmacytoid dendritic cells *via* TLR7 (17), and HIV DNA triggers CD4+ T cell pyroptosis and the release of inflammatory cytokines (18). The external envelope glycoprotein Gp120 (19) and the accessory protein Vpr (20) activate monocytes/macrophages, whereas the transmembrane envelope glycoprotein Gp40 promotes T cell activation by interacting with the T cell receptor (21). Moreover, the transactivator protein Tat has been reported to induce oxidative stress *via* NFkB in B cells (22).

Finally, even though microbial antigen-driven B cell and T cell activation is intended to be beneficial, protracted activation will result in immune senescence, decreased potential to respond to other stimuli, and immune deficiency.

Concerning the infectious agents for which we have therapeutic possibilities, the treatment of these pathogens will reduce the levels of immune activation. Yet, even virologically efficient drugs may not abolish all of the immune activation, as it has been abundantly proven for HIV infection (23).

From the point of view of benefit to the microbial agent, triggering a global and generalized activation of the immune system may be paradoxically desirable, if it is able to ultimately evade the storm of the activated immune system and co-exist with chronic activation and the resulting senescence. Yet again, HIV provides an example, whereby it evades antibody responses by constant generation of envelope variants (24), which can act as neoantigens, and continually drive germinal center reactions associated with generation of newly activated CD4 target cells (25).

In this Research Topic, the role of residual microbial load, microbial translocation, and adipose tissue alterations will be addressed.

Younas et al. have identified a particular profile of immune activation in people living with HIV-1 (PLHIV) presenting residual viral load under ART. This profile is characterized by CD4+ T cell, monocyte, and endothelial activation. These data reinforce the hypothesis that low-level viremia could drive a specific form of immune activation in virologic responders. Many studies using the single copy assay of plasma viremia have shown that most patients have persistent plasma viremia in the range of 1-10 copies/ml despite being classified as fully suppressed by standard clinical monitoring assays (26).

However, there now appears to be a spectrum of activity of the HIV proviral reservoir in patients fully suppressed on ART. A new highly sensitive assay using circulating CD4 T cells has shown that there is a 3 log_10_ range of the level of intracellular HIV RNA transcripts within different patients’ CD4 T cells (27), that has not been stopped by ART. Patients at the higher end of this range are those that can have plasma viral load blips (27), consistent with previous results that higher levels of intracellular unspliced HIV-1 RNA in PBMC from patients on ART correlated with shorter time to HIV-1 recrudescence after treatment interruption (28).

Another cause of immune activation is explored by Isnard et al., Mak et al., and Ancona et al., microbial translocation due to loss of gastrointestinal barrier integrity, as a result of HIV infection. Isnard et al. review the mechanisms of immune activation related to fungal translocation in HIV infection. They discuss the different markers of fungal translocation, in particular, β-D Glucan, a major cell wall component of most fungi, linked to disease progression and inflammation in treated and nontreated patients. They also discuss specific strategies that could be critical to reduce the contribution of fungal translation to inflammation in PLHIV.

Mak et al. aimed to directly quantitatively measure, *in vivo*, the loss of gastrointestinal barrier integrity using the technique of confocal endomicroscopy. In contrast to what was expected, they were unable to document a clear weakness in integrity of the epithelial barrier in HIV+ patients on ART, even those who commenced therapy relatively late in disease, compared to HIV-uninfected controls. Even though this was a relatively small study, it should have shown any obvious defect in gut permeability that should have been as widespread as currently believed for PLHIV, according to the literature.

Ancona et al. aimed to measure not only longitudinal changes in markers of microbial translocation and gut microbiota in PLHIV commencing ART for 24 months, but also direct analysis of microbial flora in blood. They found chronic elevation of the plasma marker of microbial translocation, sCD14, but a clinical index of gut permeability, the ratio of lactulose and mannitol in urinary excretion, showed a similar range compared to the level reported in healthy controls and treated coeliac patients, from the literature. Similarly, the level of fecal calprotectin, another clinical marker of gut permeability, after ART appeared to be within the normal range from the literature. However, while their patients’ gut microbiota showed that they maintained an apparent gut dysbiosis, as reported in many other studies, the blood microbiota suggested selective translocation of microbes, with a higher plasma abundance of the *Proteobacteria* phylum compared to intestinal abundance.

Finally, Bourgeois et al. have reviewed the role of adipose tissue in HIV-driven immune activation. The loss of muscle and fat tissue mass during untreated HIV-1 infection and then the emergence of ART-related lipodystrophy led to intensive study of adipose tissue in PLHIV. Proteins encoded by HIV-1 may directly affect adipocytes, in addition to the indirect effect of CD4 T cell infection. The lymphatic vasculature is an important part of adipose tissue, therefore the inability of ART to completely eradicate HIV-1 infection can lead to low-grade inflammation in adipose tissue, similar to obesity, with resulting co-morbidities.

Overall, low level chronic activation of the immune system in PLHIV is probably partly due to the inability of current ART to completely suppress HIV transcriptional activity and the resulting extremely low level plasma viremia, reflecting residual production of HIV-1 proteins, and even complete virions, in various tissues.

This is in addition to the compromise of immune defenses, particularly in the gastrointestinal tract, leading to the low level of systemic stimulation by the increased presence of microbial products. Other important frontline barrier systems probably also require more focused study in this regard, particularly HIV persistence in the respiratory tract (29, 30), changes in skin, such as acne following commencement of ART (31), and perturbations in the CNS (32).

## The Forms of Microbial-Induced Immune Activation

All types of immune activation have been reported in the course of infections. For the sole example of HIV, CD4+ T cell, CD8+ T cell, B cell, NK cell, dendritic cell, monocyte/macrophage, polymorphonuclear cell, complement activation, and inflammation have been observed in non-treated as well as in ART-treated patients (23).

In this Research Topic, the types of immune activation in severe COVID-19 and HIV-associated immune reconstitution inflammatory syndrome, respectively, are reviewed by Seddiki and French. They find that there are significant parallels in the two conditions, and the link appears to be amplified activation of monocytes/macrophages associated with aberrant pro-inflammatory T and B cell adaptive responses.

## The Consequences of Microbial-Induced Immune Activation

In addition to fueling non-transmissible chronic diseases, persisting immune activation may be deleterious for the immune system itself, as it may induce immune senescence and immune deficiency. The link between immune activation and non-immune restoration is explored in the work by Shive et al. A combination of persisting elevated levels of pro-inflammatory IP-10 and reduced levels of anti-inflammatory TGF-ß1 is associated with lower CD4 cell count reconstitution in PLHIV on ART, with controlled plasma viremia.

Immune activation may also impact the capability of mounting a specific immune response. Several studies explored the link between COVID-19 severity and the development of an efficient humoral immunity with contradictory results. Moreover, the question of antibodies as beneficial, neutral or harmful in SARS-CoV-2 infection remains controversial. Feng et al. assessed SARS-Cov-2 seroprevalence in 84 hospitalized patients. They observed that the antibody response against three important antigens (RBD, N and S) dynamically changed over time to reach a peak 3-4 weeks after symptom onset with a response lasting for an average of 112 days. They found that these responses were higher in patients with a severe condition.

## Microbial-Induced Immune Activation and Therapeutic Intervention

Antimicrobial therapy may reduce immune activation and its deleterious consequences. This is the case for naïve CD4+ lymphopenia induced by HCV which is reduced by direct-acting antiviral drugs, as shown by Auma et al. resulting in at least partial or full restoration of circulating naïve CD4 T cells numbers. The importance of the size of the pool of these cells for control of future infections has recently been suggested as the cause of the age-related loss of control of SARS-CoV-2 infection in older patients (33).

This is also the case for ART which diminishes HIV-driven immune activation. Yet, there are differences between ART regimens, and potentially between 1-, 2- and 3-drug regimens as discussed by van Welzen et al.

By contrast certain antimicrobial therapies may worsen immune activation. An example thereof is given by Fu et al. They report that although PEG-IFNα-2b has early antiviral effects, it later exerts an immune activation effect inducing an upregulation of CD24^+^CD38^hi^ B cells driving an immuno-suppressive program and reducing anti-virus therapeutic effects. They show that the expansion of CD24^+^CD38^hi^ B cells correlated negatively with therapeutic effects. In this context they also found CD24 to be a suitable marker to target CD24^+^CD38^hi^ cells, and demonstrate the possibility to interrupt the immunosuppressive state using an anti-CD24 antibody.

Last, Kircheis et al. propose a therapeutic strategy targeting directly the immune activation in COVID-19. Indeed, they present the proinflammatory transcription factor NF-κB as a possible therapeutic target for treatment of the cytokine storm associated with the severe forms of COVID-19. The authors used different models*, in vitro* human macrophages, H5N1 infected BALB/c mice and LPS-induced cytokines in BALB/c mice to test VL-01, a proteasome inhibitor. They demonstrated the efficacy of this inhibitor *in vitro* and in mice to significantly reduce the release of pro-inflammatory cytokines (IL-1, IL-6, TNF-α) and chemokines (MIP-1, CXCL1).

## Author Contributions

All authors listed have made a substantial, direct, and intellectual contribution to the work and approved it for publication.

## Funding

PC was supported by MSDAvenir (DS-2016-0010).

## Conflict of Interest

The authors declare that the research was conducted in the absence of any commercial or financial relationships that could be construed as a potential conflict of interest.

## Publisher’s Note

All claims expressed in this article are solely those of the authors and do not necessarily represent those of their affiliated organizations, or those of the publisher, the editors and the reviewers. Any product that may be evaluated in this article, or claim that may be made by its manufacturer, is not guaranteed or endorsed by the publisher.
